# Natural history of coronary stents: 14 year follow-up of drug eluting stents versus bare metal stents

**DOI:** 10.1016/j.ihj.2023.11.001

**Published:** 2023-11-04

**Authors:** Rohit Sunil Walse, Krishna Kumar Mohanan Nair, Ajitkumar Valaparambil, Bijulal Sasidharan, Harikrishnan Sivadasapillai, Jissa Vinoda Thulaseedharan

**Affiliations:** a Department of Cardiology, Sree Chitra Tirunal Institute for Medical Sciences and Technology, Thiruvananthapuram, Kerala, 695011, India; b Achuta Menon Centre for Health Sciences Studies, Sree Chitra Tirunal Institute for Medical Sciences and Technology, Thiruvananthapuram, Kerala, 695011, India

**Keywords:** Bare metal stent”, “Drug-eluting stent”, “In-stent restenosis”, “Natural history”, “Stent thrombosis”

## Abstract

**Background:**

Several randomized trials have shown the effectiveness of drug-eluting stents (DES) over bare metal stents (BMS) in terms of repeat revascularization at 1 year; however long term data in this context is conflicting.

**Aim:**

To assess the long term clinical outcomes after coronary artery stenting with drug-eluting stents and bare metal stents.

**Methods:**

This is a retrospective cohort study, including 100 consecutive patients with Coronary Artery Disease who underwent successful percutaneous intervention (PCI) with implantation of DES and contemporary 100 patients who underwent PCI with implantation of BMS in the years 2005 and 2006 at our center.

**Results:**

Over a median follow-up of 14 years, the primary composite outcome of major adverse cardiovascular and cerebrovascular events (MACCE) was found to be similar in both the groups [DES-37; BMS-36 (p value = 0.88)]. At 1 year of follow-up, the incidence of MACCE was significantly lower with DES group than BMS group [DES-3; BMS-10, P value = 0.04]; but the benefit was not seen at 5 years, 10 years and 14 years follow-up. The incidence of very late stent thrombosis in our study population was similar in either of the groups (p value = 0.13). Obesity and creatinine of >1.4 mg/dl were found to be the predictors of all-cause death.

**Conclusion:**

In patients with coronary artery disease, the composite endpoint of MACCE for the first year after stenting was significantly lower in patients receiving DES than those receiving BMS; however, at very long term follow-up, the event rates were similar.

## 1 Introduction

Coronary angioplasty with stent implantation is currently one of the established modalities for the invasive treatment of patients with symptomatic coronary artery disease.^1^ Within a few years after the introduction of DES, safety concerns were raised, particularly about late stent thrombosis (ST).^1^, ^2^, ^3^ In 2006, incomplete neointimal coverage was identified as the cause of such increased risk of stent thrombosis with DES, as reported from pathoanatomical studies,^4^ randomized trials^3^ and registries.^5^ Although short term trials had shown safety and efficacy of DES over BMS in terms of repeat revascularization & stent thrombosis,^6^ long-term studies reported that the lower rate of stent thrombosis with DES in comparison to BMS was limited during the first-year post-stenting, and after 1 year, DES and BMS were associated with similar ST rates.^7^^,^^8^

With the development of newer generation DES with lower strut thickness, there is improved survival benefit and reduced target vessel revascularization, especially in patients of ST segment elevation myocardial infarction, however very long-term data comparing clinical outcomes following PCI with DES and BMS is lacking. We planned to evaluate the outcomes of DES and contemporary BMS over further long-term follow-up.

## 2 Material and methods

**Fig. 1:**
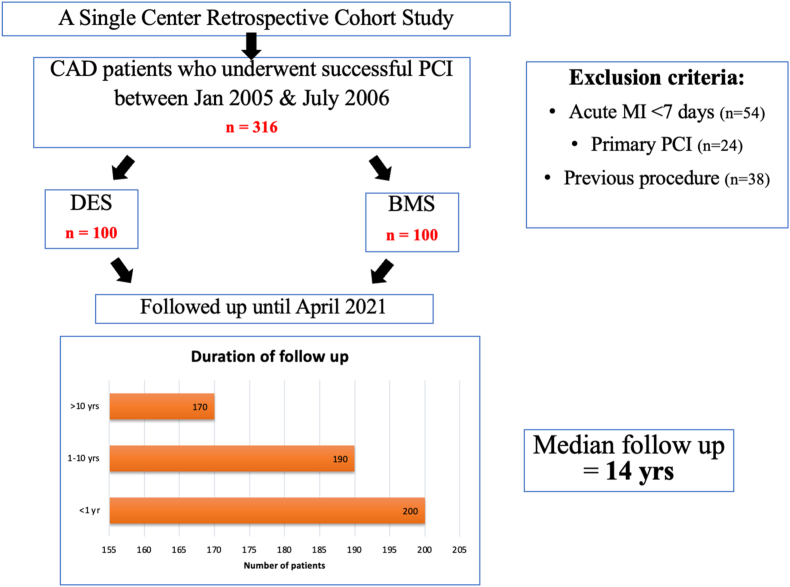
Patient flowchart (BMS-Bare metal stent, CAD-coronary artery disease, DES-Drug eluting stent, MI-Myocardial infarction, PCI-percutaneous intervention).

It was a single-centre retrospective observational study conducted at the department of Cardiology, at a tertiary care canter in India. The study protocol was approved by the Institutional Ethics Committee. We included consecutive hundred patients with coronary artery disease, who underwent successful PCI with implantation of drug-eluting stents (DES) between January 2005 to July 2006 and contemporary hundred patients who underwent PCI with implantation of bare metal stent (BMS). These patients were subsequently followed up till April 2021 [Fig. 1]. The following patients were excluded from the study^1^: patients with a history of acute (<7 days from onset) myocardial infarction,^2^ patients who underwent primary PCI,^3^ those who had any procedure done in the past (e.g. plain old balloon angioplasty {POBA}, stenting, etc).

Prospective data were entered into a database that contained demographic, clinical, angiographic and procedural information. Clinical follow-up was carried out through patient visits, telephone interviews, mailed questionnaire and medical record reviews. Follow-up data was collected with regards to total deaths (cardiovascular or non-cardiovascular), myocardial infarction (MI), in-stent restenosis (ISR), target lesion revascularization (TLR), target vessel revascularization (TVR) and stroke. The data on stent thrombosis and interventions to other vessels was also obtained. The primary composite outcome was identified as major adverse cardiovascular and cerebrovascular events (MACCE), namely all-cause death, MI, ISR, TLR, TVR, any other vessel revascularization and stroke.

The data analysis was performed using the SPSS Statistics software for Windows Version 25. Continuous variables were expressed as mean ± SD and categorical variables as proportions. Kaplan–Meier analysis was done to determine event-free survival rates. The chi-square test was used for multivariate regression analysis.

## 3 Results

We conducted a retrospective analysis including a total of 200 patients, consecutive 100 patients each receiving drug eluting stents (DES) and bare metal stents (BMS) at our institute in the year 2005–2006. This time period was intentionally chosen to eliminate initial stent-related issues and have a better comparison between the groups when both DES & BMS have been implanted in equal frequency. The Drug-eluting stents used in our population included Paclitaxel eluting stents (COSTAR, INFINIUM), Sirolimus-eluting stents (CYPHER, MAGIC, PRONOVA, S FLEX) and Zotarolimus eluting stent (ENDEAVOR). Contemporary bare metal stents (ANGSTROM, ARTHOS, DRIVER, LEKTON, PROKINETIC, PROLINK, PENTA, MINIVISION, HELI, FLEXMASTER, PIXEL, TITAN2, S-7, S-670, TSUNAMI GOLD) were used in the BMS group. The complete follow-up was available for 85 % of patients. The median follow-up duration for the study group was 14 years. Baseline clinical, angiographic and procedural characteristics are shown in [Table 1]. The risk factor profiles of the 2 groups were largely comparable.

**Table 1:** Baseline characteristics.

Baseline Characteristics n (%)	Overall (200)	DES (100)	BMS(100)	P Value
AGE (yrs)	52.42 ± 8.93	52.07 ± 9.15	52.78 ± 8.74	0.57
Gender (Male)	179 (89.5)	88	91	0.48
Obesity	29 (14.5)	16	13	0.54
DM	84 (42)	45	39	0.39
HTN	93 (46.5)	45	48	0.67
DLP	151 (75.5)	75	76	0.86
F/H/O CAD	65 (32.5)	37	28	0.17
SMOKING	102 (51)	44	58	0.048
Diagnosis				
STEMI	86 (43)	44	42	0.77
NSTEMI	51 (25.5)	27	24	0.62
CSA	63 (31.5)	29	34	0.44
Functional class				
n (%)				
-I	11 (5.5)	3	8	0.21
-II	164 (82)	85	79	0.26
-III	25 (12.5)	12	13	0.83
EF (%)	65.94 ± 10.29	66.93 ± 9.93	64.95 ± 10.59	0.17
Hb (g/dl)	14.14 ± 1.45	14.11 ± 1.52	14.17 ± 1.38	0.76
Creat (mg/dl)	1.13 ± 0.2	1.14 ± 0.2	1.12 ± 0.2	0.65
FBS (mg/dL)	123.55 ± 58.97	127.58 ± 65.73	119.28 ± 50.9	0.34
TC (mg/dL)	168.35 ± 40.69	162.34 ± 39.94	174.36 ± 40.79	0.05
TG (mg/dL)	153.78 ± 68.49	152.42 ± 67.01	155.14 ± 70.31	0.79
HDL (mg/dL)	35.81 ± 8.53	34.31 ± 7.27	37.30 ± 9.43	0.02
LDL (mg/dL)	102.61 ± 36.82	99.11 ± 37.21	106.03 ± 36.34	0.1
No. of vessel involved:				
1	108 (54)	55	53	0.77
2	73 (36.5)	34	39	0.46
3	19 (9.5)	11	8	0.46
Dominant Circulation:				
Right	175 (87.5)	85	90	0.28
Left	9 (4.5)	5	4	0.73
Co-dominant	16	10	6	0.29
Vessel Involvement:				
LAD	123 (61.5)	68	55	0.059
LCX	90 (45)	47	43	0.57
RAMUS	4	1	3	0.31
RCA	91 (45.5)	39	52	0.06
Type of lesion:				
A	76 (38)	39	37	0.77
B1	67 (33.5)	29	38	0.17
B2	39 (19.5)	17	22	0.37
C	18	15	3	0.003
Vessel Treated:				
LAD	99 (49.5)	56	43	0.066
LCX	50 (25)	27	23	0.51
RAMUS	3 (1.5)	0	3	NA
RCA	66 (33)	26	40	0.035
No. of stents:				
1	161 (80.5)	84	77	0.21
2	36 (18)	16	20	0.46
3	3 (1.5)	0	3	–

(Abbreviations: BMS-Bare metal stents, Creat- Creatinine, CSA- Chronic stable angina, DES- Drug eluting stents, DLP- Dyslipidemia, DM-Diabetes mellitus, EF- Ejection fraction, FBS-fasting blood sugar, F/H/O CAD-family history of coronary artery disease, Hb- Haemoglobin, HDL-high density lipoprotein, HTN- Hypertension, LDL-low density lipoprotein, NSTEMI- Non ST elevation myocardial infarction, STEMI- ST elevation myocardial infarction, TC- total cholesterol, TG-triglycerides).

The mean age of patients was 52.07 ± 9.15 years in DES group whereas 52.78 ± 8.74 years in BMS group (p value = 0.57). Male predominance was observed in either of the groups with higher prevalence of cardiovascular risk factors. Percentage of smokers was significantly higher in BMS group as compared to DES group (p value = 0.048). Both the groups had similar number of patients with ST elevation myocardial infarction (STEMI), non ST elevation myocardial infarction (NSTEMI) and chronic stable angina (CSA). All these patients did not have any prior procedure in the past and all the interventions done were elective. HDL cholesterol was significantly higher in BMS group when compared to DES group. Type C lesions were found to be significantly higher in DES group (p value = 0.003) and significantly higher RCA lesions were treated in BMS group (p value = 0.035).

A total of 247 stents were implanted. Mean number of stents per patient in DES group was 1.20 and in the BMS group was 1.27 (p value = 0.18). The mean length was significantly higher in the DES group than BMS group [20.42 ± 6.53 mm in DES vs 17.65 ± 3.9 mm in BMS group (p value 0.006)] [Table 2].

**Table 2:** Stent characteristics.

Stent Characteristics	Overall (247)	DES (120)	BMS(127)	P Value
Length (mm)	19.00 ± 5.51	20.42 ± 6.53	17.65 ± 3.90	0.006
Diameter (mm)	2.82 ± 0.35	2.82 ± 0.30	2.82 ± 0.39	0.81
Pressure (atm)	12.96 ± 2.34	12.90 ± 2.03	13.02 ± 2.64	0.88
Time (sec)	21.14 ± 5.30	21.14 ± 5.52	21.13 ± 5.13	0.89

(Abbreviations: BMS- Bare metal stents, DES-Drug eluting stents).

Over a median follow up of 14 years, the primary composite outcome of major adverse cardiovascular and cerebrovascular events (MACCE) was similar in both the groups [37 in DES group and 36 in BMS group; p value = 0.88)]. Individual outcome analysis revealed all-cause death, cardiovascular death, myocardial infarction, in stent restenosis, target lesion revascularization, target vessel revascularization, any other vessel revascularization and stroke were similar in both the groups. Our study did not have any incidences of acute or subacute stent thrombosis.

**Fig. 2:**
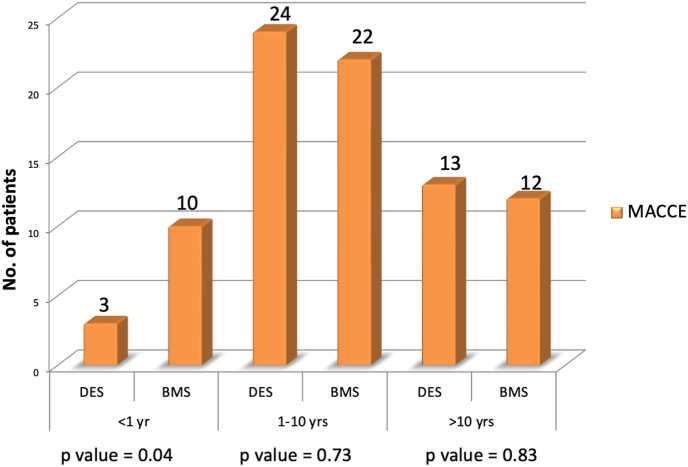
Cumulative major adverse cardiovascular and cerebrovascular events within 1 year, 1–10 years and >10 years of follow up (BMS-Bare metal stents, DES-Drug eluting stents, MACCE-major adverse cardiovascular and cerebrovascular events).

Further detailed analysis of the outcomes [Fig. 2] showed that within 1 year of follow up, the incidence of MACCE was significantly lower with DES group than BMS group [DES-3%; BMS-10 %, P value = 0.04]. Majority of the events occurred in 1–10 year follow-up period after stenting in either of the groups and were statistically insignificant.

**Fig. 3:**
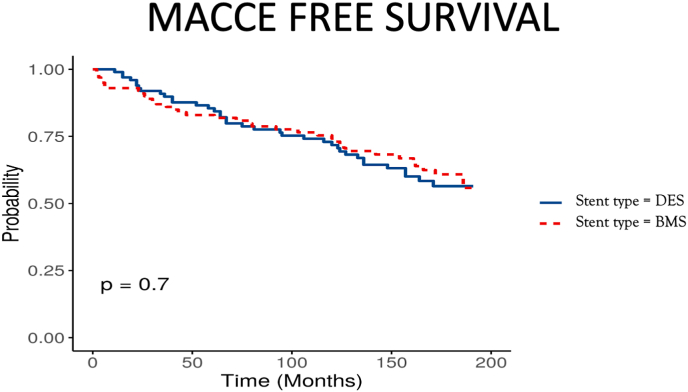
Cumulative survival free of major cardiovascular and cerebrovascular events (MACCE) (BMS-Bare metal stents, DES-Drug eluting stents).

**Fig. 4:**
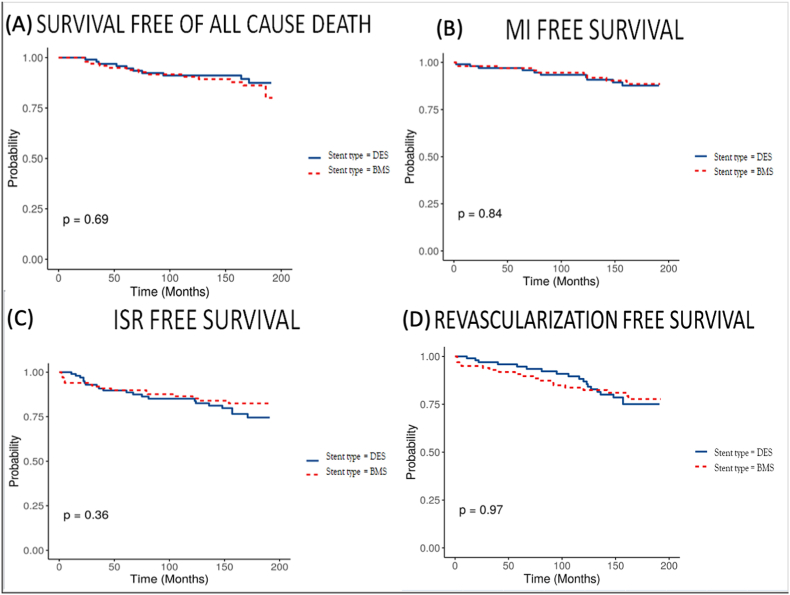
Cumulative survival free of all cause death (A), Myocardial infarction free survival (B), In stent restenosis free survival (C) and repeat revascularization free survival (D) (BMS-Bare metal stents, DES-Drug eluting stents).

Kaplan Meier analysis revealed that there were no significant difference between the two study groups in terms of MACCE [Fig. 3]. Secondary outcome analysis also showed that there were no significant differences between the two groups in terms of all cause death, myocardial infarction, in-stent restenosis and overall revascularization over a long term follow up [Fig. 4].

Predisposing factor analysis by multivariate regression showed a fasting blood sugar level of >125 mg/dl was significantly associated with MACCE (HR 1.95, 95 % CI-1.13–3.38, p value = 0.016). HDL level of >40 mg/dl was associated with lesser incidence of MACCE [HR 0.37, 95 % CI-0.15–0.89, p value = 0.028)]. Triple vessel disease was associated with higher incidence of MACCE (HR 2.74, 95 % CI-1.21–6.19, p value = 0.015). Obesity and creatinine of >1.4 mg/dl were found to be associated with higher incidence of all cause death [(HR-6.02, 95 % CI-1.92–18.52, p value = 0.002) and (HR-7.34, 95 % CI - 2.39-22.55, p value < 0.001) respectively]. An ejection fraction of >60 % was found to be protective for all cause death (HR-0.30, 95 % CI-0.11–0.76, p value = 0.012). In stent restenosis was found to be significantly higher with FBS level >125 mg/dl (HR-2.27, 95 % CI-1.12–4.57, p value = 0.022). However, an ejection fraction of >60 % was found to have lesser incidence of in stent restenosis (HR-0.42, 95 % CI-0.20–0.84, p value = 0.016).

Despite having significantly higher number of Type C lesions in DES group and significantly higher number of RCA lesions treated in BMS group, these variables were not found to be associated with higher event rates.

## 4 Discussion

After a median follow up of 14 years, we observed that the primary composite outcome of major adverse cardiovascular and cerebrovascular events (MACCE) was similar in both groups [p value = 0.88]). Further sub-analysis revealed that MACCE was significantly lower with DES group than BMS group at 1 year of follow up and subsequently became comparable during later follow up. Similar findings were reported in earlier studies.^8^^,^^9^ When compared to 10 year follow up study by Vale et al,^10^ the lower all-cause mortality rates in our study despite of a longer follow-up was probably due to younger population and exclusion of patients with prior interventions. However cardiac mortality rate in bare metal stent group was 8 % over 14 years follow up which was relatively less when compared to study by Harikrishnan et al^11^ where it was 16 % over 20 years follow-up. The similar MI rates in our study at 5 years (3 % in DES group and 3 % in BMS group), at 10 years (6 % in DES group & 5 % in BMS group [p value = 0.75]) and at total follow up were comparable and similar to NORSTENT (9) trial and EXAMINATION(12) trial.

In our study population, the rate of in-stent restenosis and stent thrombosis was observed to be similar in DES group and BMS group at the end of 14 years of total follow up. This finding was not consistent with other studies^7^^,^^8^^,^^12^ which have demonstrated benefit of drug eluting stents over bare metal stents in terms of stent thrombosis at 1 year of follow up; however NORSTENT trial^9^ demonstrated consistent benefit of drug eluting stents over bare metal stents in terms of definite stent thrombosis at 6 years of follow up. We found similar rates of overall revascularization at 14 years of follow up. This finding was different from other trials^8^^,^^9^ which have demonstrated a clear benefit of drug eluting stents over bare metal stents in terms of repeat revascularization. Lower percentage of revascularization rates was probably due to a heterogeneous population with relatively stable coronary lesions.

Our study revealed, fasting blood sugar level of >125 mg/dl at presentation and triple vessel disease are independent predictors for MACCE whereas obesity and creatinine of >1.4 mg/dl at presentation are predictors of all cause death. We also found that high fasting blood sugar level (>125 mg/dl) had a significant association with occurrence of in-stent restenosis.

In our study the benefit of drug eluting stents in terms of various event rates was probably masked due to more complex lesions in drug eluting stent group, older generation drug eluting stents with larger strut size, stainless steel platform and larger stent length leading to comparable number of events as compared to bare metal stents. However we believe that newer generation improved drug eluting stents with Cobalt chromium platform, thinner struts, biodegradable polymer would certainly prove better over bare metal stents and do not recommend reconsidering bare metal stents for clinical practice. Our study is the only study in Indian literature which serves as a reference for very long term follow-up of drug eluting stents and bare metal stents stating their natural history in patients with coronary artery disease.

## 5 Conclusions

• The composite endpoint of MACCE for the first year after stenting was significantly lower in patients receiving DES than those receiving BMS.
• During the later follow up at 5 years, 10 years and at 14 years, the event rates were similar in patients receiving DES & BMS.
• Over a long term follow up of 14 years, drug eluting stents offer no significant benefit over bare metal stents in terms of very late stent thrombosis.
• High fasting blood sugar level at presentation was found to be an independent predictor of major adverse cardiovascular and cerebrovascular events and in-stent restenosis.
• Our results may serve as the historical reference data for the long term follow-up of BMS & DES in this era of drug eluting stents.

## Declaration of competing interest

The authors declare that they have no known competing financial interests or personal relationships that could have appeared to influence the work reported in this paper.
